# Biliary Drainage During Neoadjuvant Chemotherapy in Pancreatic Cancer: Evidence and Practical Recommendations

**DOI:** 10.3390/cancers18030467

**Published:** 2026-01-30

**Authors:** Tadahisa Inoue, Masanao Nakamura, Kiyoaki Ito

**Affiliations:** Department of Gastroenterology, Aichi Medical University, 1-1 Yazakokarimata, Nagakute 480-1195, Aichi, Japan

**Keywords:** pancreatic cancer, pancreatic adenocarcinoma, neoadjuvant chemotherapy, obstructive jaundice, preoperative biliary drainage, plastic stent, self-expandable metal stent, recurrent biliary obstruction, percutaneous transhepatic biliary drainage

## Abstract

Pancreatic cancer frequently presents with obstructive jaundice resulting from distal malignant biliary obstruction. Neoadjuvant chemotherapy (NAC) is increasingly applied in resectable and borderline resectable disease. In this context, uncontrolled cholestasis or cholangitis may hinder timely chemotherapy initiation and cause unplanned hospitalizations and treatment delays; therefore, preoperative biliary drainage is essential to ensure safe and uninterrupted NAC. This review summarizes current biliary drainage strategies during NAC, focusing on key clinical goals, maintaining durable patency throughout the planned NAC course, minimizing infectious and procedure-related morbidity, reducing the need for reintervention, and avoiding adverse effects on subsequent pancreatoduodenectomy, as well as on practical decision-making in clinical practice. We compare transpapillary drainage via endoscopic retrograde cholangiopancreatography (ERCP) using plastic stents and self-expandable metal stents (SEMSs) and discuss the emerging “slim” fully covered SEMSs designed to reduce the risks of pancreatitis and cholecystitis while maintaining sufficient patency. Endoscopic ultrasound-guided biliary drainage is also reviewed as an important salvage option after failed ERCP and as a potential primary approach in selected patients, and we also discuss conventional percutaneous approaches. Overall, current evidence supports an individualized, algorithm-based strategy that prioritizes durable internal drainage to maintain NAC schedules, reserves percutaneous transhepatic biliary drainage for specific indications, and underscores the need for further prospective studies evaluating long-term surgical and oncologic outcomes in resectable disease.

## Simple Summary

Pancreatic cancer often causes jaundice by blocking the bile duct, which can delay neoadjuvant chemotherapy. Preoperative biliary drainage is therefore used to normalize bilirubin, prevent cholangitis, and avoid unplanned hospitalizations. This review summarizes evidence and practical recommendations for drainage during chemotherapy in resectable and borderline resectable disease. ERCP is typically first line. Compared with plastic stents, self-expandable metal stents usually provide longer patency and fewer reinterventions across the planned treatment course. EUS-guided drainage is an important option after failed ERCP and may be primary in selected patients, while percutaneous drainage is reserved for specific situations.

## 1. Introduction

Pancreatic cancer frequently presents with obstructive jaundice caused by distal malignant biliary obstruction. In patients with resectable or borderline resectable pancreatic cancer, neoadjuvant chemotherapy (NAC) is increasingly employed to enhance margin-negative resection rates and to manage micrometastases before surgery [1,2]. However, biliary obstruction may complicate NAC administration, as elevated bilirubin levels and cholangitis can delay or prevent timely chemotherapy initiation. Consequently, preoperative biliary drainage (PBD) has become a crucial consideration in this clinical context. Historically, the routine use of PBD in jaundiced patients scheduled for immediate surgery was controversial, with some studies suggesting it could be omitted if surgery was imminent [3]. In the NAC era, however, surgical intervention is postponed for multiple chemotherapy cycles, rendering effective biliary drainage essential to ensure safe and uninterrupted treatment [4].

NAC is increasingly used in both resectable and borderline resectable pancreatic cancer, although the intended duration to surgery often differs. Borderline resectable disease frequently requires a longer preoperative interval, which increases the importance of durable biliary drainage and minimizing unplanned reinterventions. While the core objective is the same in resectable and borderline resectable (avoiding cholangitis and treatment interruption), drainage strategies should be selected with the anticipated time-to-surgery in mind.

This review provides a comprehensive summary of biliary drainage strategies during NAC for resectable and borderline resectable pancreatic cancer with distal biliary obstruction. It discusses the rationale for PBD, compares endoscopic stenting options, examines the role of endoscopic ultrasound-guided biliary drainage (EUS-BD), and considers percutaneous approaches. Key clinical outcomes, including stent patency, dysfunction rates, reinterventions, cholangitis, effects on chemotherapy timing, hospitalizations, and surgical results, are analyzed in light of recent evidence.

## 2. Rationale for Preoperative Biliary Drainage in the NAC Setting

In patients with pancreatic head cancer undergoing NAC, relieving biliary obstruction before chemotherapy is typically necessary. Cholestasis and cholangitis not only present direct risks, such as hepatic dysfunction and sepsis, but also hinder chemotherapy administration, as many cytotoxic agents cannot be safely delivered in the presence of hyperbilirubinemia. Accordingly, current guidelines recommend PBD for any patient with significant jaundice who is scheduled to receive neoadjuvant therapy [5,6,7].

The main indications for PBD include (1) cholangitis, which constitutes an absolute indication because uncontrolled infection can delay treatment; (2) marked hyperbilirubinemia or symptomatic jaundice (e.g., pruritus, malabsorption), where drainage improves patient performance status and enables full-dose chemotherapy; and (3) an anticipated long interval before surgery, as is characteristic of NAC. Even in patients with resectable disease and moderate, asymptomatic jaundice, when surgery is delayed by several weeks or months of NAC, most centers advocate biliary decompression to prevent cholangitis and hepatic dysfunction during that period [8]. Conversely, if surgery is expected within one to two weeks, such as in pre-NAC treatment paradigms, PBD may be omitted unless cholangitis is present.

It is important to recognize that PBD itself carries risks. Historical trials involving patients who underwent immediate surgery showed no improvement in surgical outcomes with routine preoperative stenting; in fact, one landmark study reported higher complication rates when PBD was performed before pancreatoduodenectomy [3]. However, these data predate the modern NAC approach. In the NAC context, the situation changes, avoiding PBD is generally not feasible given the several-month interval before resection. The main benefit of PBD during NAC is that it enables prompt initiation and uninterrupted delivery of chemotherapy by normalizing bilirubin levels and preventing treatment interruptions due to biliary sepsis or hepatic dysfunction. A recent international consensus emphasized that PBD is generally recommended in contemporary practice for patients receiving NAC for resectable or borderline resectable pancreatic cancer, with the goal of completing therapy without delays from recurrent biliary obstruction (RBO) or cholangitis. Studies have shown that patients who experience cholangitis or chemotherapy interruptions due to biliary complications have poorer survival outcomes, underscoring the importance of maintaining biliary patency throughout NAC. Nevertheless, PBD techniques should be optimized to minimize procedure-related adverse events, such as pancreatitis or insertion-site infections, which themselves may delay chemotherapy.

In summary, the rationale for PBD before NAC is to create an opportunity for chemotherapy in patients with obstructive jaundice. The optimal PBD strategy should achieve rapid biliary decompression, remain patent throughout the entire NAC course, minimize adverse events, and avoid negative effects on subsequent surgery. The following sections review available biliary drainage modalities in relation to these objectives.

## 3. Endoscopic Transpapillary Drainage via ERCP

Endoscopic retrograde cholangiopancreatography (ERCP) with transpapillary stent placement has long been the first-line approach for managing distal malignant biliary obstruction [9]. It provides internal drainage, without the need for an external catheter, and preserves normal anatomy, which is advantageous before surgery. In patients with pancreatic cancer undergoing NAC, ERCP-based drainage is generally attempted unless contraindications are present (e.g., an inaccessible papilla due to altered surgical anatomy or duodenal obstruction). When ERCP is performed for preoperative drainage, the key consideration is the type of stent to use. The two main options are plastic stents (PSs) and self-expandable metal stents (SEMSs), each with distinct characteristics.

***Plastic stents (PSs)***: These are typically 7–10 mm French polymer endoprostheses. They are inexpensive and easy to place or replace. However, PSs have a limited internal diameter (approximately 2–3 mm) and tend to occlude within several weeks to a few months, mainly due to sludge accumulation and bacterial biofilm formation. During an extended NAC course (often lasting two to four months or longer), a single PS rarely maintains patency for the entire duration. Multiple studies have reported very high rates of stent occlusion and cholangitis associated with PS use in NAC patients [3,10]. Such events necessitate frequent reinterventions (e.g., stent exchanges), which may interrupt chemotherapy. Therefore, PSs may be suitable for short-term drainage or palliative cases requiring only a brief interval of biliary decompression but is suboptimal for patients receiving NAC.

***Self-expandable metal stents (SEMSs)***: These stents have a substantially larger diameter (8–10 mm when fully expanded) and demonstrate markedly longer patency than PSs. Fully covered SEMSs are generally preferred in the preoperative setting because they can be removed during surgery and reduce the risk of tumor ingrowth. However, a randomized trial comparing covered and uncovered SEMSs for biliary drainage during NAC reported no significant differences in major clinical outcomes, including RBO and cholangitis [11]. A SEMS allows bile to flow through a wider lumen, greatly reducing the likelihood of occlusion due to sludge formation. Multiple randomized controlled trials and meta-analyses have demonstrated that SEMSs outperform PSs in maintaining biliary drainage throughout NAC [12,13,14,15] (Table 1). The most recent meta-analysis by Lyu et al. (2023) showed that SEMSs were associated with significantly lower rates of reintervention (OR 0.04, 95% CI 0.01–0.10), RBO (OR 0.09, 95% CI 0.02–0.40), and cholangitis (OR 0.16, 95% CI 0.03–0.79) compared with PSs (*p* < 0.05 for all) [15]. Moreover, SEMSs were associated with fewer delays of neoadjuvant therapy (OR 0.17, 95% CI 0.05–0.61), and pooled RBO rates were 19.88% with SEMSs versus 57.53% with PSs, reflecting greater stent reliability. Importantly, these meta-analyses and clinical studies found no significant difference in postoperative complication rates between SEMSs and PSs. This finding alleviates earlier concerns that metal stents might increase surgical complexity or infection risk; in practice, postoperative outcomes, including R0 resection rates and overall morbidity, appear comparable regardless of the stent type used. Based on this consistent evidence, current guidelines recommend SEMSs for PBD in patients with pancreatic cancer undergoing NAC [6,7,9,16]. The superior patency of SEMSs enables completion of NAC without the need for multiple ERCPs or unplanned reinterventions.

Given these advantages, SEMSs have become the preferred option in many centers for patients undergoing NAC. Furthermore, a randomized controlled trial by Gardner et al. [12] demonstrated clear clinical benefits: patients assigned to the SEMS group experienced substantially fewer occlusion events than those with PSs, which also resulted in lower overall hospitalization costs. Similarly, a multicenter study reported that SEMSs were more cost-effective in the neoadjuvant setting, as their longer patency reduced the need for repeat procedures despite higher initial costs [17].

***Stent-Related Adverse Events***: Although SEMSs clearly reduce the risk of occlusion, they have a distinct profile of adverse events that warrants consideration. Placement of a covered SEMS across the papilla can occasionally obstruct the cystic duct or pancreatic duct orifices. Acute cholecystitis is a recognized complication when the covered stent blocks cystic duct outflow, although this occurs in only a small proportion of cases (approximately 5–10%) [18]. Acute pancreatitis may also develop after ERCP, particularly if the pancreatic duct is inadvertently obstructed or contrast is injected. Covered SEMSs have been associated with a higher incidence of post-ERCP pancreatitis (PEP) compared with PSs [14,19,20], likely because the larger device traverses the papilla and may impair pancreatic duct drainage [21]. Performing a minor sphincterotomy before SEMS placement has been proposed as a strategy to reduce the risk of post-ERCP pancreatitis; however, evidence remains inconclusive, and several studies have failed to demonstrate a consistent protective effect, rendering this approach controversial [22]. The observed pancreatitis rate is generally low (about 5%), and with meticulous technique, the risk of post-ERCP pancreatitis associated with modern covered SEMSs appears acceptable, although it remains an important clinical consideration. In contrast, PSs, being smaller in diameter, tend to cause less papillary trauma. Post-ERCP pancreatitis can still occur with PS placement, but the stent itself rarely obstructs the pancreatic duct.

A growing concern in the preoperative setting is whether the larger caliber of SEMSs contributes to increased rates of cholecystitis or pancreatitis. The proximal flare of a 10 mm SEMS may cover the cystic duct orifice, potentially leading to gallbladder distension and secondary infection. These observations have prompted interest in refining SEMS design for use in NAC, as discussed in the following section.

## 4. 10 mm Standard vs. 6 mm “Slim” Metal Stents

To balance the objectives of maximizing stent patency while minimizing adverse events, recent investigations have explored the use of smaller-caliber SEMSs. A 6 mm fully covered SEMS (often referred to as a “slim” SEMS) has been introduced and evaluated in this context [23,24,25]. Intuitively, a 6 mm stent has roughly half the cross-sectional area of a 10 mm stent yet remains approximately twice the diameter of a standard PS. The underlying hypothesis is that a 6 mm covered SEMS may provide adequate biliary drainage during NAC, as its diameter corresponds to approximately 18 French, much larger than any PS, while potentially reducing the risk of occluding side ducts, such as the pancreatic and cystic ducts. The smaller profile may also adhere less to the bile duct wall, facilitating easier stent removal and minimizing tissue trauma. Indeed, 6 mm covered SEMSs have been reported to exhibit a trend toward lower rates of cholecystitis and post-ERCP pancreatitis compared with 10 mm stents (cholecystitis: 3.0% vs. 11.8%; post-ERCP pancreatitis: 3.6% vs. 10.5%, respectively) [23] (cholecystitis: 1.7% vs. 13.6%; post-ERCP pancreatitis: 5.1% vs. 8.5%, respectively) [26]. However, other studies have found no significant differences in clinical outcomes or adverse event rates between the two stent sizes (e.g., RBO: 39% vs. 23%, and overall stent-related AEs: 4% vs. 15%, for 6 mm vs. 10 mm, respectively) [27], while some have described higher migration rates and more frequent RBO with 6 mm SEMS (migration: 26.9% vs. 0%; 90-day RBO: 30.8% vs. 3.8%, for 6 mm vs. 10 mm, respectively) [28]. Therefore, the overall clinical utility of 6 mm covered SEMSs remains unproven. As all current evidence derives from retrospective studies, the results of the ongoing STARDOM trial (jRCT1042250093), which directly compares 10 mm covered SEMSs, 6 mm covered SEMSs, and PSs for PBD during neoadjuvant therapy, are highly anticipated.

In summary, ERCP with stent placement remains the first-line approach for managing distal biliary obstruction in patients with resectable or borderline resectable pancreatic cancer. SEMSs are generally preferred over PSs in those receiving NAC because of their superior durability and longer patency. PSs may still be appropriate for short-term drainage or when cost and access limitations exist; however, elective stent exchanges should be anticipated. Emerging evidence on 6 mm fully covered SEMSs suggests that these stents can maintain adequate biliary drainage with potentially fewer adverse effects, although further prospective studies are needed to confirm these findings. During NAC, careful monitoring is essential, and if an ERCP-placed stent becomes occluded, prompt reintervention should be performed to maintain treatment continuity.

## 5. EUS-Guided Biliary Drainage as a Salvage or Primary Strategy

When ERCP fails or is not feasible, EUS-BD provides an internal drainage alternative to surgical or percutaneous approaches. Over the past decade, EUS-BD has evolved from an experimental technique into an increasingly accepted rescue therapy for malignant biliary obstruction [29,30]. Procedures such as EUS-guided choledochoduodenostomy (EUS-CDS) and EUS-guided hepaticogastrostomy (EUS-HGS) enable direct access to the biliary tree using a fine needle under endoscopic ultrasound guidance, followed by stent placement from the bile duct into the gastrointestinal lumen, thereby completely bypassing the papilla. For distal biliary obstructions, such as those associated with pancreatic head cancer, EUS-CDS is the preferred technique. This involves puncturing the extrahepatic bile duct from the duodenal bulb and deploying either a SEMS or a lumen-apposing metal stent (LAMS) between the bile duct and duodenum. Alternatively, when duodenal access is limited or obstructed, EUS-HGS can be performed to drain the left intrahepatic ducts into the stomach.

***Salvage after ERCP failure***: When ERCP cannot be performed, for instance, due to a tight malignant duodenal stenosis preventing endoscope passage or an inaccessible papilla resulting from ampullary tumor involvement, EUS-BD serves as an effective salvage option. It eliminates the need for an external drain and can often be performed during the same session or shortly after a failed ERCP. In experienced centers, the technical success rate of EUS-BD approaches over 90%, and clinical success, defined as effective biliary decompression, is similarly high, exceeding 90% in recent reports [31]. Common adverse events associated with EUS-BD include bile leakage into the peritoneal cavity, bleeding, and stent malposition; these occur in approximately 10–15% of cases [32], although most can be managed conservatively. Importantly, EUS-BD provides internal drainage, thereby obviating the need for an external catheter. This typically results in improved patient comfort and quality of life compared with percutaneous drainage.

***Role as primary drainage***: Some experts now advocate considering EUS-BD as a primary drainage approach in selected patients. Two recently published randomized controlled trials compared EUS-CDS using a LAMS with conventional ERCP employing a SEMS as primary therapy for malignant distal biliary obstruction, primarily in unresectable cases [33,34]. These studies found no significant differences in overall clinical success, stent patency duration, or adverse event rates between the two techniques, although procedure time was shorter with EUS-CDS. While those trials largely enrolled palliative patients, they demonstrated that EUS-BD is not inferior to ERCP. Furthermore, there is a theoretical rationale for favoring EUS-HGS in potentially resectable patients, as the transmural tract and stent are positioned outside the surgical field of pancreaticoduodenectomy [35].

For patients with resectable disease, ERCP remains the standard first-line drainage method, particularly because it preserves native anatomy. However, when ERCP is expected to be technically challenging, some centers may opt for upfront EUS-BD as an alternative strategy. For EUS-BD, outcome estimates vary substantially because many reports include predominantly palliative populations; therefore, rates should be interpreted with caution when extrapolating to NAC candidates. Where reported in cohorts proceeding to pancreatoduodenectomy, preoperative EUS-BD did not appear to compromise surgical outcomes, although NAC-specific prospective data remain limited.

***Impact on Subsequent Surgery***: A critical question is whether creating a new biliary–enteric fistula through EUS-BD adversely affects later pancreatoduodenectomy. Although data remain limited, available evidence is generally reassuring. Several small case series and reports have documented successful pancreatoduodenectomy following EUS-BD, with the EUS-placed stents removed either endoscopically before surgery or intraoperatively [36,37,38]. A French multicenter cohort study compared surgical outcomes in patients who underwent EUS-CDS versus transpapillary stenting prior to pancreatoduodenectomy and found fewer surgical complications with EUS-CDS, along with no significant differences in oncologic outcomes [37]. Likewise, multiple reports have described successful pancreatoduodenectomy after EUS-HGS, indicating that the procedure can be performed safely with meticulous surgical technique [39,40]. Another recent study assessing primary EUS-HGS performed before pancreatoduodenectomy observed no postoperative disadvantages, reporting no significant differences among patients who underwent no biliary drainage, ERCP stenting, or EUS-HGS in terms of major postoperative adverse events or positive peritoneal lavage cytology rates [35].

Overall, current evidence suggests that EUS-BD does not compromise the feasibility or outcomes of curative surgery. This finding is noteworthy, as earlier skepticism regarding EUS-BD in resectable disease largely stemmed from concerns about complicating the surgical field. With the advent of dedicated removable devices, such as LAMS, and increasing procedural expertise, these concerns are gradually diminishing. Nevertheless, robust prospective data in resectable or NAC populations are still lacking, and the long-term implications of creating a transluminal tract, including potential effects on adhesions, biliary reconstruction, and oncologic outcomes, remain uncertain. Therefore, EUS-BD should be pursued cautiously and ideally within high-volume centers, while further prospective validation is awaited.

***When to Use EUS-BD***: In clinical practice, EUS-BD is most commonly employed following a failed ERCP during NAC evaluation. A typical scenario involves a patient with duodenal obstruction caused by a pancreatic tumor in whom the endoscopist cannot reach or cannulate the papilla. Instead of resorting to percutaneous drainage, an EUS-BD can be performed to achieve internal biliary drainage. This approach spares the patient the discomfort and inconvenience of an external drain and can often be performed during the same session. EUS-BD may also be advantageous in patients with concurrent biliary and gastric outlet obstruction. In such cases, a single EUS session can be used to place both a biliary stent and, when indicated, a duodenal stent or perform an EUS-guided gastrojejunostomy. However, operator expertise remains a critical determinant of success and safety. EUS-BD should be undertaken only by advanced endoscopists experienced with the procedure, as improper puncture or stent placement can result in serious adverse events.

In summary, EUS-BD has become an essential component of the therapeutic armamentarium for patients undergoing NAC. It serves as an effective salvage technique after ERCP failure, achieving high rates of internal biliary drainage without compromising subsequent surgery. Increasingly, EUS-BD is incorporated into PBD algorithms, particularly in high-volume centers with the necessary expertise. Future studies and ongoing clinical trials will further define its role, including its potential as a primary rather than backup drainage modality. At present, the practical approach can be summarized as follows: ERCP should be attempted first, and if unsuccessful, EUS-BD is the preferred next step, over percutaneous drainage, in institutions experienced with this technique.

## 6. Percutaneous Transhepatic Biliary Drainage

Percutaneous transhepatic biliary drainage (PTBD) has long served as a reliable method for relieving biliary obstruction and remains an important fallback option when endoscopic internal drainage is not feasible. In this procedure, an interventional radiologist introduces a catheter percutaneously through the liver into a bile duct, typically under combined ultrasound and fluoroscopic guidance. The clinician may either maintain an external drainage catheter or advance an internal stent across the obstructed segment. PTBD allows for rapid decompression of the biliary system even in anatomically complex cases and does not depend on endoscopic access to the duodenum.

However, PTBD presents several disadvantages in the preoperative setting. Most notably, it leaves an external drain that the patient must manage throughout the course of NAC. The external catheter, often connected to a bile collection bag, can substantially affect quality of life, as patients must cope with daily tube care, the risk of accidental dislodgement, and restrictions on physical activity. Furthermore, external drainage carries a persistent risk of infection; PTBD catheters are prone to bacterial colonization and may lead to catheter tract infections or cholangitis if occlusion occurs. Routine dressing changes and catheter flushes are therefore necessary. From a therapeutic standpoint, continuous external bile loss can cause fluid and electrolyte depletion, necessitating careful monitoring to prevent dehydration and malabsorption during chemotherapy.

Additional Considerations: Percutaneous drains also carry a risk of tumor seeding along the catheter tract. Because the needle and catheter traverse both the hepatic parenchyma and abdominal wall, they may dislodge tumor cells that subsequently implant along the drainage pathway. In cholangiocarcinoma, this phenomenon is well-documented and poses a challenge to achieving surgical curability [41]. Although tumor seeding from PTBD in pancreatic cancer is less common, several cases have been reported [42]. A 2019 systematic review indicated that PTBD is associated with a higher incidence of metastatic seeding compared with endoscopic stenting in patients with resectable malignancies [43]. While this risk remains relatively low, many surgeons prefer to avoid PTBD before potentially curative surgery whenever possible to minimize the chance of tract recurrence.

PTBD can also complicate the surgical field. Because the drain traverses the liver, the catheter tract may require excision or cauterization during surgery to prevent postoperative bile leakage. In some cases, surgeons resect a small portion of liver surrounding the catheter insertion site if there is concern for tumor seeding.

***When to Utilize PTBD***: PTBD should be considered when endoscopic approaches are unavailable or unsuccessful, and EUS-BD is not feasible. For example, in centers without EUS-BD capability or when an attempted EUS-BD also fails, PTBD remains the final option to relieve biliary obstruction. PTBD may also be appropriate for patients who cannot undergo endoscopy because of comorbid conditions or when the duodenum is completely obstructed, making EUS access technically challenging or excessively risky. Once a PTBD catheter is established, certain patients may subsequently be converted to internal drainage. Through the existing percutaneous access, a SEMS can be deployed across the obstruction to achieve internal biliary drainage. If technically successful, the external catheter can sometimes be removed, leaving an internal metal stent bridging the obstruction, functionally equivalent to an endoscopic stent. This approach, known as percutaneous transhepatic biliary stenting, can improve quality of life by eliminating the external drainage component, although it necessitates an additional procedure. In such cases, the external drainage bag is typically transient, and some disadvantages of prolonged external drainage may be mitigated.

During NAC, if a PTBD catheter remains in place, close coordination between the oncology and interventional radiology teams is essential to ensure continuous catheter function throughout treatment. Obstruction of the drainage catheter can rapidly precipitate cholangitis and delay chemotherapy. To prevent such complications, regular catheter exchanges, typically every 4 to 6 weeks, are often scheduled proactively. Despite these logistical challenges, PTBD remains highly effective for achieving biliary decompression, as virtually any patient with patent intrahepatic ducts can be drained via this route. In terms of patency, an external drainage catheter does not “occlude” in the same manner as a stent, since bile will continue to flow as long as the external system remains open. Functional problems generally occur when the catheter becomes clogged; these are often managed with saline flushing or by upsizing the catheter to restore patency. Quantitative NAC-specific data for PTBD remain limited; nevertheless, given the burden of external catheter care and infection-related concerns, PTBD is best reserved for situations in which endoscopic internal drainage is not feasible, with conversion to internal drainage considered whenever possible.

***Impact on NAC***: Patients with PTBD can generally receive full-dose chemotherapy, and in some cases, external bile drainage may even prevent transient bilirubin elevations due to tumor lysis. The primary impact of PTBD during NAC involves patient comfort and the potential for treatment disruption due to drain-related complications. Vigilance is essential for early recognition and management of drain-associated infections, which should be treated promptly to prevent chemotherapy interruption. Comparative studies have reported that patients with PTBD experience more frequent unplanned interventions and occasional treatment delays compared with those who have internal biliary stents, reinforcing the general preference for internal drainage methods [44,45].

In summary, PTBD serves as a valuable backup for biliary drainage during NAC when endoscopic stenting cannot be performed. It provides reliable jaundice relief and enables chemotherapy to proceed safely. However, its disadvantages include the presence of an external drainage apparatus and a higher risk of infection and patient discomfort. Whenever possible, internal drainage via ERCP or EUS-BD is preferred for improved patient quality of life and potentially lower adverse event rates. PTBD should therefore be reserved for cases in which endoscopic options are unavailable or have failed.

## 7. Key Takeaways and Conclusions

Table 2 summarizes the principal features of the different biliary drainage strategies used in the NAC setting. The adoption of neoadjuvant therapy for pancreatic cancer has introduced new requirements for biliary drainage techniques. The primary objective is to provide reliable, long-term biliary decompression that endures through several months of chemotherapy and potentially chemoradiation, while minimizing the need for reintervention and reducing the risk of adverse events that could interrupt oncologic treatment. If RBO occurs during NAC, prompt reintervention is recommended to restore drainage and minimize chemotherapy interruption.

In patients with resectable or borderline resectable pancreatic cancer complicated by distal biliary obstruction, preoperative biliary drainage is generally indicated before initiating NAC, except in rare instances of very mild, asymptomatic jaundice associated with a short NAC regimen. Current evidence suggests endoscopic stenting with a SEMS as the preferred initial drainage method in these patients. Compared with conventional PSs, 10 mm covered SEMSs provide markedly superior patency, significantly lower rates of cholangitis, and, consequently, a reduced risk of chemotherapy interruption. This enhanced drainage durability increases the likelihood of completing NAC as scheduled, which may in turn improve oncologic outcomes. Importantly, these advantages are achieved without compromising surgical safety: multiple studies and meta-analyses have confirmed that the preoperative use of SEMSs does not adversely affect the success or complication rates of subsequent pancreatoduodenectomy; however, more rigorous prospective evaluation remains warranted. More recently, emerging stent concepts, such as 6 mm diameter multi-hole SEMSs, have been proposed to potentially reduce ERCP-related adverse events, but clinical evidence remains limited. These devices should be considered investigational in the NAC setting pending prospective studies.

That said, the choice of drainage strategy should be individualized; one approach does not suit every patient in this complex population. Key considerations include patient anatomy (for instance, whether the endoscope can reach the papilla or whether a duodenal obstruction necessitates alternative access), clinical condition (whether the patient can tolerate ERCP or if PTBD under conscious sedation would be safer), local expertise (availability of EUS-BD as a salvage option and the level of endoscopist proficiency in advanced techniques), and economic or resource-related factors (in some regions, repeated PSs may still be practiced due to financial limitations, albeit with close follow-up). Context-specific optimization remains essential. For example, in a patient with duodenal tumor invasion, a center with strong EUS expertise may proceed directly to EUS-BD rather than attempt multiple ERCPs, thereby avoiding procedural delays and added risk. Conversely, a patient with an easily accessible papilla and elevated bilirubin can often be managed expeditiously with ERCP and SEMS placement, which typically ensures durable drainage throughout NAC. In high-volume institutions proficient in EUS, a practical protocol may involve attempting ERCP once, followed by immediate “Plan B” EUS-BD within the same session if cannulation fails, thus securing internal drainage without resorting to PTBD. Another important nuance involves patients who initially received a PS, often before referral: many experts recommend upgrading to a metal stent prior to commencing NAC to minimize the risk of mid-therapy stent occlusion and treatment interruption.

Additionally, quality of life remains an often underappreciated but important consideration. Internal endoscopic drainage via ERCP or EUS-BD spares patients the discomfort, inconvenience, and lifestyle restrictions associated with external catheters, a factor of particular relevance during NAC when patients are already managing treatment-related adverse effects. Whenever feasible, internal drainage should therefore be prioritized for its positive impact on patient well-being. In contrast, PTBD, although effective as backup options, should be reserved as last-line interventions during the NAC period because of their greater effect on patient comfort and higher risk of complications that may disrupt chemotherapy.

Looking ahead, ongoing innovations continue to hold promise for further reducing adverse events such as pancreatitis and cholangitis, potentially making preoperative biliary drainage even safer and more streamlined. Moreover, multidisciplinary coordination among gastroenterologists, surgeons, and oncologists is essential to promptly address any biliary complications that arise during NAC, ensuring patients remain on schedule for surgery. Effective communication is particularly critical, for example, if a patient presents for chemotherapy with fever and right-upper-quadrant pain, an immediate gastroenterology consultation to evaluate possible stent occlusion can prevent a minor issue from escalating into a significant treatment delay.

In conclusion, the optimal biliary drainage strategy during NAC for pancreatic cancer is one that maximizes stent patency and minimizes morbidity, thereby enabling the neoadjuvant regimen to proceed as planned and allowing the patient to reach curative surgery in optimal condition. For most patients, this entails ERCP with placement of a fully covered SEMS as first-line therapy. When standard ERCP is not feasible, EUS-BD serves as an excellent salvage approach that maintains internal drainage. Percutaneous drains remain important components of the management algorithm but should be reserved for situations in which endoscopic techniques are unsuccessful or unavailable. By tailoring the drainage approach to each patient’s anatomical and clinical context and leveraging advances in endoscopic stenting, clinicians can deliver NAC with minimal interruption, an outcome that ultimately enhances tumor downstaging, R0 resection rates, and long-term survival. The overarching principle is to relieve biliary obstruction in the least disruptive and most physiologically appropriate manner, keeping the primary focus on oncologic therapy. With careful planning and collaborative, multidisciplinary care, biliary drainage during NAC can be optimized to support, rather than hinder, the patient’s path toward curative surgery and beyond.

## Figures and Tables

**Table 1:** Key Studies Evaluating Biliary Stenting Strategies During Neoadjuvant Therapy for Pancreatic Cancer.

Study (Year)	Design/Setting	Population	Comparison	Key Biliary Outcomes	Surgery-Related Outcomes	Key NAC/Surgery-Relevant Message
Gardner et al. (2016) [12]	RCT	PC on NAT	10 mm CSEMS vs. 10 mm USEMS vs. PS	CSEMS demonstrated longer time to occlusion	Attempted surgical resection rates equivalent	CSEMS associated with fewer NAC delays; cost-effectiveness comparable
Seo et al. (2019) [11]	RCT	PC on NAT	8/10 mm CSEMS vs. 8/10-mm USEMS	No significant difference in clinical success, stent patency, or AEs	Curative-intent surgery rates and operative difficulty similar between groups	Both covered and uncovered SEMSs acceptable; selection should consider removability and risk of tumor ingrowth
Tamura et al. (2021) [13]	RCT	BR PC on NAC	10 mm CSEMS vs. PS	CSEMS showed fewer RBO and reintervention events	No significant difference in postoperative AEs	CSEMS better suited for the NAC course than PS
Lyu et al. (2023) [15]	Meta-analysis	PC on NAT	SEMS vs. PS	SEMS associated with reduced RBO and reintervention rates	No significant differences in postoperative complications, bile leakage, or R0 resection rates	SEMS supports uninterrupted NAC compared with PS
Kumar et al. (2023) [14]	Meta-analysis	PC on NAT or CRT	SEMS vs. PS	SEMS demonstrated better patency and fewer stent-related AEs	R0 resection and postoperative complication rates equivalent	SEMS minimized treatment interruptions

AE, adverse event; BR, borderline resectable; CSEMS, covered self-expandable metal stent; CRT, chemoradiotherapy; NAC, neoadjuvant chemotherapy; NAT, neoadjuvant therapy; PC, pancreatic cancer; PS, plastic stent; RBO, recurrent biliary obstruction; RCT, randomized controlled trial; SEMS, self-expandable metal stent; USEMS, uncovered self-expandable metal stent.

**Table 2:** Comparison of Biliary Drainage Strategies During Neoadjuvant Chemotherapy.

Strategy	Patency & RBO	Reinterventions	AEs (Biliary-Specific)	Impact on NAC	Impact on Surgery & QOL
Endoscopic Plastic Stent	Short patency; high rate of RBO within 1–2 months	Planned exchanges every 4–8 weeks; frequent unscheduled ERCPs	Cholangitis common; PEP possible (~5% per ERCP)	Frequent interruptions or delays due to clogging or cholangitis	Internal drainage; easy removal at surgery; frequent interventions negatively affect QOL
Endoscopic Metal Stent (10 mm CSEMS)	Long patency; low RBO rates throughout typical NAC	Usually none; reintervention only if RBO or stent-related event occurs	Low cholangitis rate; PEP ~5%; cholecystitis ~5%; stent migration ~5%	Fewer biliary events, enabling uninterrupted NAC; no scheduled exchanges required	Internal drainage; typically removable during surgery; minimal impact on surgical field
“Slim” Metal Stent (6 mm CSEMS)	Generally good patency; RBO rates low to moderate (data heterogeneous)	Usually none; possible increase if RBO/stent-related events occur (conflicting evidence)	Some studies show lower rates of cholecystitis and PEP; others report higher migration or RBO rates	Promising for maintaining uninterrupted NAC, though evidence remains inconsistent	Internal drainage; removal feasible; clinical role not yet established (limited to retrospective data)
EUS-Guided Biliary Drainage (EUS-CDS or EUS-HGS)	High patency during NAC; durable internal drainage tract	Typically none; reintervention if RBO occurs	Low cholangitis rate; bile leakage, abscess, or bleeding possible; pancreatitis rare	Generally allows for continuation of NAC; delays occur mainly if EUS-related AEs develop	Internal drainage; CDS stent usually resected with specimen; HGS tract outside surgical field; requires high procedural expertise
Percutaneous Transhepatic Biliary Drainage	Effective while catheter remains patent; requires maintenance	Routine tube exchanges every 4–6 weeks; dislodgement or occlusion relatively common	Cholangitis and catheter-site infections possible; bleeding and tract seeding rare but reported	NAC can proceed if patient stable, but infections or revisions may cause treatment delay; higher procedural burden	External drainage device reduces QOL; risk of tract-related issues and tumor implantation; surgery feasible but with higher infection risk

AE, adverse event; CDS, choledochoduodenostomy; ERCP, endoscopic retrograde cholangiopancreatography; EUS, endoscopic ultrasound; CSEMS, covered self-expandable metal stent; HGS, hepaticogastrostomy; NAC, neoadjuvant chemotherapy; PEP, post-ERCP pancreatitis; QOL, quality of life; RBO, recurrent biliary obstruction.

## Data Availability

No new data were created or analyzed in this study.
